# Draft genome sequence of *Streptomyces* sp. OP7 and *Streptomyces harbinensis* OP7 strains isolated from *Zea mays* and *Helianthus annuus* rhizosphere soil

**DOI:** 10.1128/mra.01110-24

**Published:** 2025-06-09

**Authors:** Olubukola O. Babalola, Oghoye P. Oyedoh, Ayansina S. Ayangbenro

**Affiliations:** 1 Food Security and Safety Focus Area, Faculty of Natural and Agricultural Sciences, North-West University 274472 , Mmabatho, South Africa; 2 Imperial College London, Silwood Park Campus 98991, Ascot, United Kingdom; University of Maryland School of Medicine, Baltimore, Maryland, USA

**Keywords:** actinomycetes, fungal pathogens, antiSMASH, inhibitory activity, metabolites

## Abstract

The genomic characteristics of *Streptomyces* sp. OP7 and *Streptomyces harbinensis* OP7 strains isolated from the rhizosphere soil of *Zea mays* and *Helianthus annuus* after harvest are presented. The *in silico* metabolites produced by these strains are reported to have antagonistic effects against fungal pathogens of food crops.

## ANNOUNCEMENT

Metabolites from Actinomycetes have gained interest as sustainable biotechnological products that are applicable predominantly in the pharmaceutical industry (1), with less focus on their agricultural benefit. Hence, this study was designed to select antagonistic Actinomycetes strains active against plant pathogens, which could be applied as potential phytoprotective *Actinomycetes* from the rhizosphere soil of food crops.

The rhizosphere soil samples were randomly selected (25°47′26.5ʺN 25°37′04.1ʺE: maize and 25°47′26-3ʺS 25°37′04.3ʺE: sunflower), uprooted, and 20 g of soil tightly adhering to the roots was collected (2). One gram of each soil sample was serially diluted with sterile distilled water and plated on Actinomycetes Isolation Agar (AIA) at 30°C, pH 7, for 3 days. Pure and discrete colonies of the strains were purified on AIA (Condalab, Spain) after two iterations under the same growth conditions. Pure cultures on AIA under the same conditions were used for DNA extraction following the kit’s protocol with a Fungal/Bacterial DNA MiniPrep Extraction Kit (Zymo Research, USA). Genome sequencing of isolates *Streptomyces* sp. OP7 and *Streptomyces harbinensis* OP7 was done at Novogene Co. Ltd., Singapore. The Illumina Nextera DNA Flex library preparation kit was used to prepare the paired-end sequencing library from the DNA samples. The PE Illumina library was loaded onto the NovaSeq 6000 (2 × 150 bp) instrument for cluster generation and sequencing to produce 7,892,750 and 5,864,399 pairs of reads, respectively.

The raw reads were trimmed using the Trimmomatic software version 0.36 (3). Reads were assembled using the SPAde assembler version 3.15.3, and the qualities of the assemblage were confirmed using CheckM version 1.0.18 (4). The genomes were annotated using Rasttk version 1.073 (5), and the taxonomy of the genomes was classified as *Streptomyces* sp. OP7 and *Streptomyces harbinensis* OP7, with an ANI of 95.96 and 98.87, respectively, using GTDB-Tk version 2.3.4 (6). The FASTA file of the genome was blasted on the CRISPR-CasFinder web server to detect the CRISPR array (Table 1) (7) and antiSMASH software version 7.0.1 (8) to identify the biosynthetic genes and the most similar metabolites coded by the genes. Each core biosynthetic gene per region was blasted on UniProt software (9) and NCBI (blastp) to confirm the key biosynthetic genes expressed by the antiSMASH server (with all extra features turned on). Default settings were used for all bioinformatics tools.

**TABLE 1:** Genomic characteristics of both strains

Genomic features	*Streptomyces* sp. OP7	*Streptomyces harbinensis OP7*
Based on contigs		
Contig sequence size	7,109,246	5,858,402
Number of contigs	37	42
GC content	72.4	73
*N*_50_ value	563,188	212,727
*L*_50_ value	5	9
Genes	6,452	5,229
Protein coding regions	6,624	5,202
tRNA	66	138
Number of subsystems	315	282
mRNA	64	5,208
Other genes	3	0
Pseudogenes	113	0
CRISPR regions	19	13
Genomic features	6,688	5,248

The *in silico* metabolite profiling of *Streptomyces* sp. OP7 and *Streptomyces harbinensis* OP7 strains revealed the presence of agriculturally important metabolites, which include terpenes (geosmin, lycopene, albaflavenone, and carotenoid), alkylresorcinol, lanthipeptides, butyrolactone, polyoxypeptin, ectoine, and desferrioxamine B and E, and naringenin (10, 11). Therefore, these strains could act as phytoprotective strains of agricultural biotechnology relevance for direct use as bioinoculants or chemocontrol metabolites against fungal pathogens of the crops.

## Data Availability

*Streptomyces* sp. OP7 and *Streptomyces harbinensis* OP7’s whole genome project has been deposited at DDBJ/ENA/GenBank under accession JBEGWG000000000 (https://www.ncbi.nlm.nih.gov/datasets/genome/GCF_040203675.1/) and JBEWTE000000000 (https://www.ncbi.nlm.nih.gov/datasets/genome/GCA_040537935.1/), respectively. The versions described in this paper are versions JBEGWG010000000 and JBEWTE010000000, with the raw reads available under BioSample and BioProject accession numbers SAMN41058886 and SAMN42050044, and PRJNA1103719 and PRJNA1128385, respectively. The strains have sequence reads archived under the accession numbers SRR28795958 and SRP516134, respectively.
